# Current Status of Neurofeedback for Post-traumatic Stress Disorder: A Systematic Review and the Possibility of Decoded Neurofeedback

**DOI:** 10.3389/fnhum.2019.00233

**Published:** 2019-07-17

**Authors:** Toshinori Chiba, Tetsufumi Kanazawa, Ai Koizumi, Kentarou Ide, Vincent Taschereau-Dumouchel, Shuken Boku, Akitoyo Hishimoto, Miyako Shirakawa, Ichiro Sora, Hakwan Lau, Hiroshi Yoneda, Mitsuo Kawato

**Affiliations:** ^1^Computational Neuroscience Laboratories, Department of Decoded Neurofeedback, Advanced Telecommunications Research Institute International, Kyoto, Japan; ^2^Department of Psychiatry, Graduate School of Medicine, Kobe University, Kobe, Japan; ^3^Department of Neuropsychiatry, Osaka Medical College, Osaka, Japan; ^4^The Florey Institute of Neuroscience and Mental Health, Melbourne, VIC, Australia; ^5^Sony Computer Science Laboratories, Inc., Tokyo, Japan; ^6^Flower of Light Clinic for Mind and Body, Tokyo, Japan; ^7^Department of Psychology, University of California, Los Angeles, Los Angeles, CA, United States; ^8^Department of Brain Research Institute, University of California, Los Angeles, Los Angeles, CA, United States; ^9^Department of Psychology, University of Hong Kong, Pokfulam, Hong Kong; ^10^RIKEN Center for Advanced Intelligence Project (AIP), Tokyo, Japan

**Keywords:** PTSD, real-time functional magnetic resonance imaging, multi-voxel decoding, fMRI decoded neurofeedback (DecNef), neural reinforcement, neuromodulation

## Abstract

**Background:** Post-traumatic stress disorder (PTSD) is a neuropsychiatric affective disorder that can develop after traumatic life-events. Exposure-based therapy is currently one of the most effective treatments for PTSD. However, exposure to traumatic stimuli is so aversive that a significant number of patients drop-out of therapy during the course of treatment. Among various attempts to develop novel therapies that bypass such aversiveness, neurofeedback appears promising. With neurofeedback, patients can unconsciously self-regulate brain activity via real-time monitoring and feedback of the EEG or fMRI signals. With conventional neurofeedback methods, however, it is difficult to induce neural representation related to specific trauma because the feedback is based on the neural signals averaged within specific brain areas. To overcome this difficulty, novel neurofeedback approaches such as Decoded Neurofeedback (DecNef) might prove helpful. Instead of the average BOLD signals, DecNef allows patients to implicitly regulate multivariate voxel patterns of the BOLD signals related with feared stimuli. As such, DecNef effects are postulated to derive either from exposure or counter-conditioning, or some combination of both. Although the exact mechanism is not yet fully understood. DecNef has been successfully applied to reduce fear responses induced either by fear-conditioned or phobic stimuli among non-clinical participants.

**Methods:** Follows the Preferred Reporting Items for Systematic Reviews and Meta-Analyses (PRISMA) guidelines, a systematic review was conducted to compare DecNef effect with those of conventional EEG/fMRI-based neurofeedback on PTSD amelioration. To elucidate the possible mechanisms of DecNef on fear reduction, we mathematically modeled the effects of exposure-based and counter conditioning separately and applied it to the data obtained from past DecNef studies. Finally, we conducted DecNef on four PTSD patients. Here, we review recent advances in application of neurofeedback to PTSD treatments, including the DecNef. This review is intended to be informative for neuroscientists in general as well as practitioners planning to use neurofeedback as a therapeutic strategy for PTSD.

**Results:** Our mathematical model suggested that exposure is the key component for DecNef effects in the past studies. Following DecNef a significant reduction of PTSD severity was observed. This effect was comparable to those reported for conventional neurofeedback approach.

**Conclusions:** Although a much larger number of participants will be needed in future, DecNef could be a promising therapy that bypasses the unpleasantness of conscious exposure associated with conventional therapies for fear related disorders, including PTSD.

## Introduction

Post-traumatic stress disorder (PTSD) is a debilitating condition following life-threatening traumatic events. PTSD is characterized by four symptom clusters, namely, re-experiencing of the traumatic event, avoidance of trauma-related stimuli, general changes in mood and cognition, and hyperarousal (DSM-5). While exposure therapy is one of the most effective treatments for PTSD (Foa and Kozak, 1986; Schnurr et al., 2007), it involves exposure to trauma-related stimuli and is itself an excruciating process. In exchange for its effectiveness, the distress of exposure therapy renders the patients with difficulties in engagement and with a considerable rate of early drop-out (i.e., 20–40% within the first 2 months of the treatment period), which may lead to suboptimal outcomes (Hembree et al., 2003; Schnurr et al., 2007). Furthermore, another limitation of exposure therapy is that 30–50% of PTSD patients do not respond to this treatment (Bradley et al., 2005). Therefore, a novel therapy for PTSD is necessary from a clinical perspective.

Neurofeedback is a promising alternative approach to ameliorate PTSD symptoms without unnecessary distress. Neurofeedback can modulate brain activity via real-time monitoring and feedback of EEG or fMRI signals, which are used to self-regulate brain functions. Repeatedly induced PTSD-related brain activity during feedback session may change its frequency of spontaneous appearance after feedback session (Kluetsch et al., 2014; van der Kolk et al., 2016). As reviewed in this article, the conventional neurofeedback mainly regulates the average EEG or fMRI signals from specific brain region in a univariate way: either up- or down-regulate the average activity of a specific region. So far, these effects are promising, but are yet to replace conventional therapy. Decoded Neurofeedback (DecNef) has recently grown rapidly as a novel neurofeedback procedure for clinical applications (Watanabe et al., 2017; Shibata et al., 2018). Instead of the average fMRI BOLD signals, DecNef allows patients to implicitly regulate multivariate voxel patterns of BOLD signals which has been decoded in advance. By targeting the multivariate patterns representing feared stimuli, DecNef has been shown to change symptom-related brain activity in subclinical phobia (Taschereau-Dumouchel et al., in submission).

Since DecNef regulates multivariate brain activity, it has three advantages over the conventional univariate neurofeedback. First, DecNef can regulate neural representation for specific stimuli, which allows one to design neurofeedback to directly intervene them. This particularly benefits the treatment of PTSD, since traumatic episodes and the related neural representations differ across individual patients. Second, it allows patients to induce ideal brain activation patterns which are likely to be observed during or after an effective exposure therapy. This might especially benefit the exposure therapy-resistant patients. For example, using a method called hyperalignment (Haxby et al., 2011; Taschereau-Dumouchel et al., 2018), the exposure therapy-resistant patients may learn to induce the neural representations which would be observed following successful exposure therapy, when such representations are inferred from the “surrogate” therapy responders. Third, DecNef can infer the causality of brain activity pattern associated with PTSD (Watanabe et al., 2017). The change of PTSD causative brain activities should change PTSD-related behavior. If DecNef only changes brain activity without affecting behavior, the seemingly PTSD causative brain activities might not be really causative. It might just be observed as a confounder: it might arise as a result of other true causative brain activity. In this regard, DecNef allows one to carefully test whether the targeted changes in brain activity accompanies the intended changes in behavior.

Despite such advantages of DecNef, whether DecNef is effective on actual PTSD symptoms is yet to be determined. To determine the future direction to developing neurofeedback for PTSD therapy, it is essential to compare the effects across different neurofeedback strategies: EEG, fMRI neurofeedback, and DecNef. Furthermore, to efficiently develop a novel treatment method based on DecNef, it is desirable to understand the exact mechanism underlying its effects.

In this review, we first discuss recent challenges in application of both EEG and fMRI neurofeedback to PTSD treatment as well as state-of-art technique that can be applied to PTSD. Second, we illustrate the potential and power of fMRI-based neurofeedback methods for PTSD treatment including DecNef. Thirdly, we discuss the possible mechanisms of DecNef on fear reduction. We hope that this review will aid the researchers who try to develop novel neurofeedback therapy on PTSD by selecting the most promising strategy among EEG or fMRI, or DecNef.

## Materials and Methods

### Systematic Literature Search

**Figure 1:**
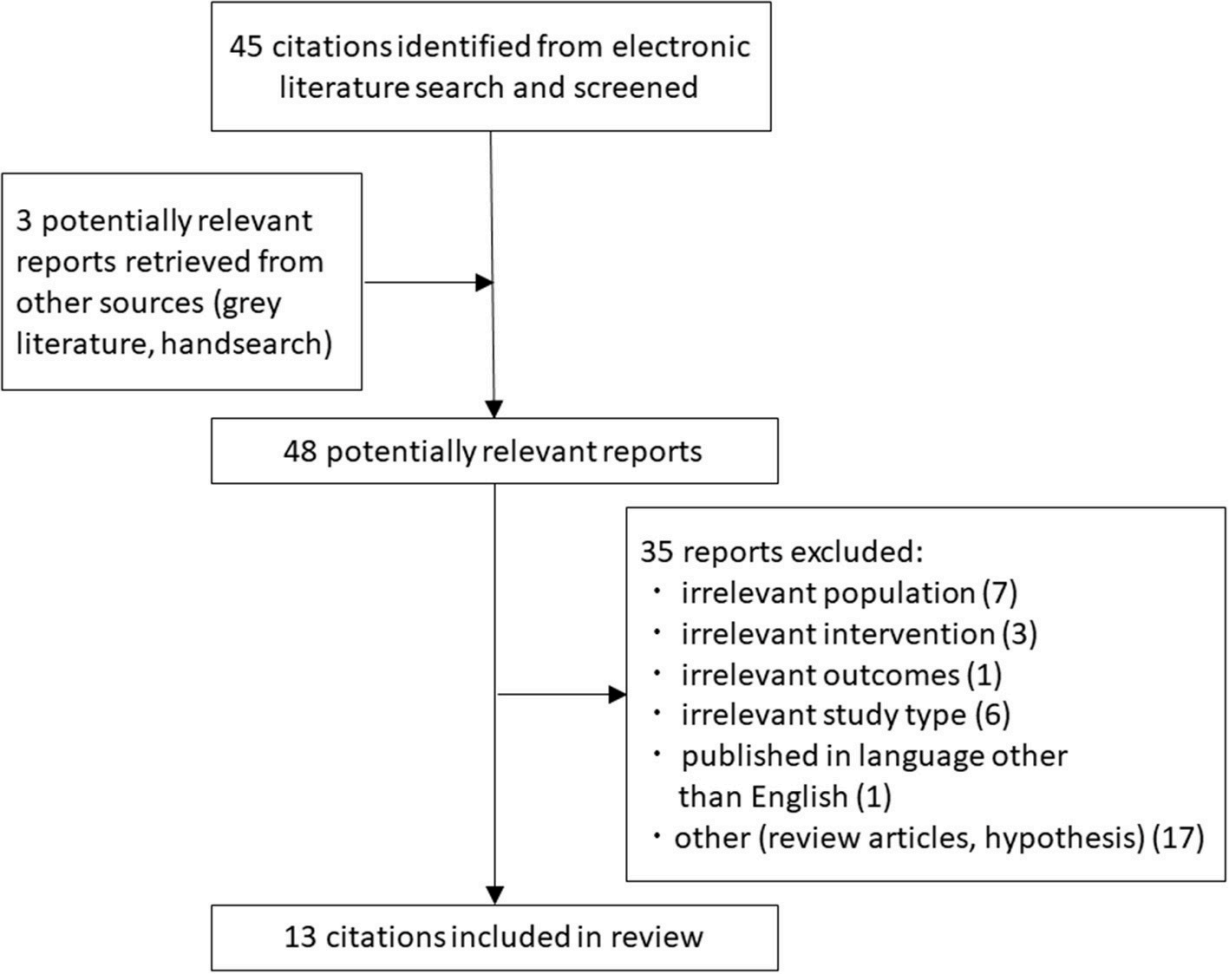
PRISMA flow-chart illustrating the results of the search strategy.

A systematic literature search was undertaken in line with the search conducted by Reiter et al. (2016). Briefly, the PubMed, PsychoInfo, and Cochrane databases were used on dates between October 5 and October 24, 2018. The following keywords were used in our search: “Neurofeedback” OR “EEG biofeedback” OR “neurotherapy” combined by AND with “PTSD” OR “post-traumatic stress disorder.” Case studies were excluded. The present systematic review follows the Preferred Reporting Items for Systematic Reviews and Meta-Analyses (PRISMA) guidelines. The inclusion criteria are presented in the PRISMA flow chart (Figure 1). Neurofeedback trials were included if they fulfilled the following criteria: (1) PTSD patients according to relevant classification systems (e.g., DSM-IV/V or ICD-10), (2) published in English, (3) comparing EEG or fMRI neurofeedback effects with regard to (a) pre vs. post-neurofeedback interventions, (b) neurofeedback vs. waiting list, (c) neurofeedback vs. sham/active neurofeedback, and (d) neurofeedback vs. conventional treatment. (4) Trials had to report (a) symptom severity or (b) brain activity at the time of the follow up. Here, a participant assigned to waiting list receives intervention after the active treatment group. In sham feedback, participants are provided with brain signal of another participant or with an artificially generated signal. In active neurofeedback, participants are provided with feedback of an alternative aspect of brain function. Titles and abstracts were screened for eligibility by one assessor (TC) (screening phase, *n* = 48). All studies not excluded in this process were examined in detail on a full text and included in this review independently by two assessors (KI, TC; *n* = 13). All reference lists of review papers and potentially eligible studies were reviewed to identify any additional papers. The risk of bias in each study was assessed by the Oxford Centre for Evidence-based Medicine, Levels of Evidence (Ellis et al., 1995). We additionally review the state-of-art studies derived from hand search during the systematic literature search.

### Decoded Neurofeedback for PTSD

We conducted a DecNef experiment for 4 individuals with PTSD with approval from the Ethics Committee of Osaka Medical College. Signed, informed consent was obtained before all procedures. Inclusion criteria were: diagnosed with DSM-IV PTSD as determined by the Clinician-Administered PTSD Scale, age of 20–55 years, traumatized by angry human males (i.e., they are victims of domestic violence or child abuse), having strong fear for passive viewing of angry face picture, which was confirmed with a score of >60 on the self-report subjective units of distress (SUDs). SUDs scale is continuum from 0 (no stress) to 100 (maximum load), and 50 is regarded as strong but barely endurable load. Exclusion criteria were: moderate or severe head injury, and/or a current diagnosis of psychosis or active suicidality in addition to general contraindication to MRI. Participants were scanned in a 3T MRI scanner (Prisma, Siemens) with a head coil at the ATR Brain Activation Imaging Center. fMRI signals were acquired using a gradient EPI sequence. During the experiments, we obtained 33 contiguous slices (TR = 2 sec, voxel size = 3 × 3 × 3.5 mm^3^, 0 mm slice gap) oriented parallel to the AC-PC plane, which covered the entire brain. We also obtained T1-weighted MR images (MP-RAGE; 256 slices, voxel size = 1 × 1 × 1 mm^3^, 0 mm slice gap).

#### Session for Decoder Construction

We first conducted a decoding session to quantify neural representations of traumatic stimuli, i.e., angry male-face pictures. The decoder was constructed so as to classify the fMRI bold signal pattern in superior temporal sulcus (STS) evoked by angry male faces from those evoked by happy female faces. Here, STS is known to represent facial emotions (Peelen et al., 2010). A modified continuous flash suppression (CFS) method was applied to render face presentation subjectively less distressing. The whole experiment comprised of 88 trials of each condition and was subdivided in 11 runs of 5 min duration. Whole exemplars (i.e., 16 exemplars) were shown once in each run in a randomized order. The obtained BOLD signals were preprocessed with mrVista software developed at Stanford University (http://vistalab.stanford.edu/software/). The functional images went through 3D motion correction without spatial and temporal smoothing. Then, the images went through rigid-body transformations to be aligned to the structural image for each participant. The BOLD signals from only the gray matter were extracted using a gray matter mask. Following preprocessing, the BOLD signals from the STS was further processed in the following steps: After removing a linear trend, the time-course in each voxel was z-score transformed within each run to minimize the baseline differences across the runs. The BOLD signal was averaged across 3 TRs which corresponded to the image presentation period at the maximum contrast (6 s). The signals were shifted by 6 s (3TRs) to compensate for the hemodynamic delay. The preprocessed fMRI signals from the STS were then used to construct a decoder to classify the activation patterns for angry vs. neutral faces. We used sparse logistic regression (SLR) (Yamashita et al., 2008) to automatically select the voxels that were relevant for classification. We trained the decoder using 176 data points obtained from 176 trials (across all 11 fMRI runs). The decoder was used in the following DecNef training to evaluate the trial-by-trial likelihood that participants could induce brain activation patterns for the angry faces.

#### DecNef Session

DecNef was conducted for 3 consecutive days following previous procedures (Koizumi et al., 2016; Taschereau-Dumouchel et al., 2018). During the DecNef training stage of our experiment, STS neural patterns of activity related to angry male faces occurred repeatedly, without the participants' awareness of their doing so. Such, successful activation of this multi-voxel pattern was reinforced with monetary reward. On each day, participants went through 11 fMRI runs with 15 trials each (20 sec per trial). Each trial had a sequence of an induction period (6 s), a fixation period (7 s), a feedback period (1 s), and an inter-trial interval (6 s). Participants were instructed to “somehow” regulate their brain activity during induction period so as to maximize the feedback score. Feedback was calculated based on how similar the induced neural pattern was to that related to angry faces. Feedback was presented as a size of a disc after 6 s of the fixation period following the induction period. A hemodynamic delay of 6 s was taken into account. Participants were not informed as to what the feedback score represented (that is, likelihood of angry face activation in the STS). The size of the disc was determined as follow: First, the functional images obtained from Induction period underwent 3D motion correction with the Turbo BrainVoyager software (Brain innovation). Second, we extracted the time-course of BOLD signals from the voxels selected during decoder construction (see decoder construction), and shifted the signals by 3TRs (i.e., 6 s) to adjust for the hemodynamic delay. Third, after removing a linear trend, the BOLD signal time-course was z-score transformed for each voxel using the BOLD signals obtained during the 20 s period following the initial 10 s period from each fMRI run. Fourth, the processed BOLD signals for each voxel were averaged across the 3 TRs corresponding to the induction period from each trial. Lastly, we calculated the likelihood that the patterns of averaged BOLD signals represented angry faces using a decoder constructed with the data from decoder construction session. The disc size (i.e., radius) was proportional to the calculated likelihood of angry faces (0–100%). The feedback disc was presented inside a ring with 5° radius, which indicated the possible maximum size of the disc. After each run, texts were presented on the monitor to inform the amount of monetary reward earned from the current run as well as the accumulated amount from all the completed runs on that day. The reward corresponded to the sum of trial-by-trial likelihoods of angry faces, scaled to yield maximum amount of 300 yen (US $2.5) per run. After completing DecNef training each day, participants received the total monetary reward in cash.

### The Mechanism of Decoded Neurofeedback

We hypothesized that the DecNef effects on fear reduction were either exposure-based (EB) or depend more on counter conditioning (CC), two common fear reduction effects achieved with the behavioral procedures to present feared objects alone without aversive outcome or to associate feared objects with positive outcome, respectively (Dickinson and Dearing, 1979; Foa and Kozak, 1986). To clarify the mechanism underlying DecNef, we mathematically modeled the effects of EB and CC separately, on the basis of the Rescorla-Wagner model (Rescorla and Wagner, 1972), and synaptic plasticity rules (Hebb, 1949). Based on this framework, we re-analyzed data from Koizumi et al. (2016) and Taschereau-Dumouchel et al. (2018).

#### Rescorla-Wagner Model

In the Rescorla-Wagner model, degree of learning is quantified in terms of associations between conditioned (CS) and unconditioned (US) stimuli. Here, CS usually means emotionally neutral stimuli which will be paired with (CS+: target stimuli) or not paired with (CS–: control stimuli) US in the fear conditioning session. US is itself aversive stimuli such as pain or loud noise. After presented with US, CS+ presentation alone would evoke fear response, which are not observed before paired with US. This model casts the conditioning processes into discrete trials, during which stimuli may be either present or absent. This model defines Δ*V*_*X*_ as the change in the strength of the association between the CS (labeled “X”) and the US:

$$\begin{array}{l} \Delta V_{\text{X}}^{n+1}=\alpha_{X}\beta \left(\lambda -{\;V}_{tot}\right)\\ {\text{V}}_{\text{X}}^{n+1}=V_{\text{X}}^{n}+\Delta V_{X}^{n+1} \end{array}$$

where α is the salience of X, β is the learning rate parameter for the US, λ is the maximum conditioning possible for the US, and Vtot is the total associative strength of all stimuli present, that is, X plus any others. That is, (λ−*V*_*tot*_) indicates the prediction error for the US. Vx is the current associative strength of X and is used to predict the associative strength of the next trial $V_{X}^{n+1}$using the expected change in the association $\Delta V_{X}^{n+\;1}$.

#### Estimation of Effect Based on Simple Exposure

In exposure-based therapy, $V_{X}^{n}$ can be considered as prediction error while α_*X*_ can be considered as likelihood for the target stimuli during induction period. Overall, part of $\Delta V_{X}^{n+1}$ results from EB effect is calculated as follows:

$$\begin{array}{lll} \Delta V_{X\left(EB\right)}^{n+1} & = & -\beta \text{ threshold}\left(L_{target\left(n\right)}-L_{control\left(n\right)}\right){\;V}_{X}^{n}\\ \Delta V_{X\left(EB\right)}^{n+1} & = & -\beta_{sp}\text{ threshold}\left(L_{target\left(n\right)}-L_{control\left(n\right)}\right){\;V}_{X}^{n} \end{array}$$

where the *threshold(X)* = *X* if *X* > 0, and 0 otherwise. The β_*sp*_ is the parameter for synaptic plasticity, that is, the learning rate of conditioning with positive value. *L*_*target*(*n*)_ is the likelihood for the target information at the *n-th* trial, while *L*_*control*(*n*)_ is the likelihood for the control information at the same trial. The extinction learning generally occurs after repeated exposure (Milosevic and Radomsky, 2008; Maren et al., 2013), therefore the expected change in the association $\Delta V_{X}^{n+1}$ through single exposure trial is postulated to be small in comparison with the strength of the association *V*_*x*_. According to this postulation, $V_{X}^{n}$ can be approximated to be constant throughout the session. Given the linear decrease in *V*_*x*_ across exposure therapy (Milosevic and Radomsky, 2008), we also assumed that the Δ*V*_*X*(*EB*)_ across trials are almost constant when the likelihood is higher than the chance level. Thus, the equation above can be approximated as follows:

$$\Delta V_{X(EB)}^{n+1}=-{\beta^{\prime }}_{\;sp}H(L_{target(n)}-L_{control\left(n\right)})$$

*H(X)* is the Heaviside step function, which is 1 if *X* > 0 and 0 otherwise. Overall, to estimate EB in line with Rescorla-Wagner model, we assumed that EB effect is linearly proportional to the total number of trials in which induction of brain activation pattern resemble the one of the target stimuli. The trial was defined as successful when likelihood of brain activation pattern for target is higher than chance level, that is, higher than 50% in Koizumi et al. (2016), and higher than the likelihood for the control animal category in Taschereau-Dumouchel et al. (2018). Thus, DecNef effect based on EB throughout the session is approximated as follows:

$$\sum_{i}\Delta V_{X\left(EB\right)}^{i}=-{\beta^{\prime }}_{\;sp}\sum_{i}H({\text{L}}_{\text{target}\left(\text{i}\right)}-{\text{L}}_{\text{control}\left(\text{i}\right)})$$

#### Estimation of Effect Based on Counter Conditioning

Regarding the CC effect, the difference between Reward and $V_{X}^{n}$ can be considered as prediction error. To estimate CC, we assumed that the trial has a fear reduction effect when the brain activation pattern for target was associated with a reward. The target brain activity is assumed to be induced when the likelihood of brain activation pattern for target was higher than chance level; i.e., 50%. We also assumed that the CC effect is a product of the two factors, namely success in induction of the neural activity pattern for the target stimuli and the amount of the reward. Because both factors are in proportion to likelihood for target pattern, the part of $\Delta V_{X}^{n+1}$ resulting from CC effect is calculated as follows:

$$\begin{array}{l} \Delta {\text{V}}_{\text{X}\left(\text{CC}\right)}^{\text{n}+1}=-\beta_{1}\;threshold\left({\text{L}}_{\text{target}\left(\text{n}\right)}-0.5\right)\left(\text{Reward}-{\text{V}}_{\text{X}}^{n}\right) \end{array}$$

where Reward is κ* threshold*(*L*_*target*(*n*)_−0.5). The κ is a coefficient of the reward. Under the assumption that *V*_*X*_ is much smaller than Reward, the equation above can be approximated as follows:

$$\begin{array}{l} \Delta {\text{V}}_{\text{X}\left(\text{CC}\right)}^{\text{n}+1}=-\beta_{1}\kappa \;threshold{\left({\text{L}}_{\text{target}\left(\text{n}\right)}-0.5\right)}^{\wedge 2} \end{array}$$

Thus, DecNef effect derived from CC throughout the session is calculated as follows:

$$\begin{array}{l} \underset{i}{\sum }\Delta V_{X\left(CC\right)}^{i}=-\beta_{1}\kappa \;\underset{i}{\sum }threshold{\left({\text{L}}_{\text{target}\left(\text{i}\right)}-0.5\right)}^{\wedge 2} \end{array}$$

#### Separate Estimation of the Effects by EB and CC

Finally, to separately estimate the effect of EB and CC on fear reduction, we assumed that the DecNef effect is weighted linear summation of *V*_*X*(*EB*)_ and *V*_*X*(*CC*)_ using mixed effect model to adjust the clustering from study type, that is either experimentally conditioned fear (Koizumi et al., 2016) or naturalistic animal phobia (Taschereau-Dumouchel et al., 2018). The mixed effect was used to adjust the difference in strength between the experimental vs. natural association with fear. Tests for absence of influential data points and independence did not reveal any violation of the assumptions for mixed effect models. The total effect is given as follows:

$$\begin{array}{l} V_{X(amg)}=\beta_{EB}\;V_{X\left(EB\right)}{}^{\prime }+\beta_{cc}\;V_{X\left(CC\right)}{}^{\prime }+\;(1|\text{paper})\\ V_{X(EB)}{}^{\prime }=V_{X\left(EB\;\right)}/\beta_{sp}{}^{\prime }\\ V_{X(CC)}{}^{\prime }=V_{X\left(EB\;\right)}/\beta_{1}\kappa \end{array}$$

where *V*_*X*(*amg*)_ is a subtraction of amygdala response to control stimuli at post-DecNef from those to target stimuli at post-DecNef. The β_*EB*_ and β_*CC*_ is the coefficient of EB effect and CC effect, respectively.

## Results

Thirteen published articles were identified that met the criteria for this review. Ten studies adopted the EEG neurofeedback approach, while 3 studies adopted the fMRI neurofeedback approach.

### Neurofeedback

#### EEG Based Neurofeedback on PTSD

EEG neurofeedback was performed to alter the power spectrum of certain filtered frequencies of activity. In line with that for other anxiety disorders (Hammond, 2005a,b, 2011; Schoenberg and David, 2014), EEG neurofeedback for PTSD is mainly used to regulate the power of either alpha waves alone or of both alpha and theta waves. Alpha activity is targeted because it is generally associated with a calm, relaxed state. PTSD patients have both decreased power and accelerated frequency of the alpha rhythm (Jokić-begić and Begić, 2003; Wahbeh and Oken, 2013). Six studies were designed to up-regulate the power of alpha rhythms either by combining rewards with alpha wave (Gapen et al., 2016; van der Kolk et al., 2016; Askovic et al., 2017) or by alpha desynchronization (Kluetsch et al., 2014; Nicholson et al., 2016; Ros et al., 2017). Alpha/theta training has been adopted in three studies (Peniston and Kulkosky, 1991; Peniston et al., 1993; Smith, 2008). Contrary to typical EEG neurofeedback for PTSD which targets alpha and/or theta waves, several studies have instead adopted sensorimotor rhythm (SMR) training (Pop-Jordanova and Zorcec, 2004; Askovic et al., 2017). SMR training was associated with enhanced attention performance and less motor activity (Sterman, 1996; Egner and Gruzelier, 2001). In one of these studies (Askovic et al., 2017), the therapists selected a neurofeedback protocol to specifically target each individual's specific maladaptive EEG patterns. Probably the most reliable empirical evidence for the success of EEG neurofeedback for PTSD came out from a study, reported above (van der Kolk et al., 2016), that was performed in the randomized, waitlist-controlled manner (van der Kolk et al., 2016). In this study, individuals with chronic PTSD in the neurofeedback group, compared with the control group, showed significant PTSD symptom improvement, as well as improvement in affect regulation capacities as measured by the Inventory of Altered Self-Capacities.

#### fMRI-Based Neurofeedback on PTSD

Conventional fMRI neurofeedback for PTSD was mainly used for modulation of amygdala activity levels (Table 1). Two studies downregulated amygdala activity during symptom provocation (Gerin et al., 2016; Nicholson et al., 2017a,b), while one study upregulated amygdala activity during happy emotion induction (Zotev et al., 2011). In one of these studies (Gerin et al., 2016), 2 of 3 patients had clinically meaningful improvement in PTSD severity as measured by CAPS, while the third patient had almost no improvement. In another of these studies (Zotev et al., 2011), a consummate technique called emotion regulation was used. In this technique, participants learn to upregulate their amygdala activity while recalling happy autobiographical memories. This technique was originally developed in the research field on depression, in which it was found to show sizable effects with a double-blind placebo control design (Young et al., 2017). In Zotev's PTSD study, however, the effect was found modest.

**Table 1:** Applications of neurofeedback for PTSD patients.

	**References**	**Sample**	**Design**							
		***N***	**%male**	**Age (years)**	**Medicated (yes/no)**	**Randomized (yes/no)**	**NF approach**	**Control group**	**Risk of bias**	**Outcome measures and measures used**
DecNef	Chiba, this manuscript	4	0	40 (mean)	Yes, *n =* 3	No	Multivariate pattern for angry face	No	C	CAPS:97.8–>54.5
f-MRI-nf	Zotev et al., 2018	23 (15 NF vs. 8 sham)	100		30.8 vs. 36.8 (mean)	Yes	Amygdala upregulation during a happy emotion induction	sham	B	CAPS: 55–>41
	Nicholson et al., 2017a,b	10	40	49.6 (mean)	Yes, *n =* 9	No	Amygdala downregulation	No	C	A shift in amygdala complex connectivity
	Gerin et al., 2016	3	100	37.3 (mean)	Yes, *n =* 3	No	Amygdala downregulation	No	C	CAPS: 65–>37
EEG-nf	Askovic et al., 2017	2	100	31(mean)	Yes, *n* = 2	No	Enhance either the SMR or alpha rhythm	No	C	HTQ:3.15–>1.85 HSCL-D: 3.30>2.1 HSCL-A: 3.2–>1.95
	van der Kolk et al., 2016	28	89	46 (mean)	Yes, *n =* 16 (NF) *n =* 10 (WL)	Yes	Enhance alpha activity	WL	B	NF: CAPS:80.1–> 44.1 DTS:67.3–>55.7 WL: CAPS:75.2–> 65.8 DTS:63.0–>60.6
	Nicholson et al., 2016	21	14	39.9 (mean)	Yes, *n =* 11;	No	Alpha desynchronization		C	A shift in amygdala complex connectivity
	Ros et al., 2017	21	14	39.9 (mean)	Yes, *n =* 11;	No	Alpha desynchronization	No	C	Decrease in TAC correlated with increases in Hurst exponent at the feedback channel Increase in Alpha amplitude
	Gapen et al., 2016	17	12	32–64 (Range)	Yes, *n =*	Yes (T4-P4 or T3-T4)	Enhance alpha activity	Active		DTS: 69.14–>49.26
	Kluetsch et al., 2014	21	14	39.9 (mean)	Yes, *n* = 11; no, *n* = 10	No	Alpha desynchronization	No	C	A shift in functional connectivity
	Smith, 2008	10	100	26–63 (Range)	Yes, *n =* 3	No	Two phased: (1) bipolar uptraining (15–18 Hz and 12–15 Hz) + theta (4–7 Hz) suppression and (2) alpha/theta (5–8 Hz) training followed by bipolar uptraining	No	C	PTSD induced symptoms of depression and attention measured by HAMD and TOVA
	Pop-Jordanova and Zorcec, 2004	10	70	9 (mean)	No	No	SMR	No	C	Skin electric resistance Brainwave changes PTSD symptoms
	Peniston et al., 1993	20	100	37.2 (mean)	Not reported	No	Alpha/theta	No	C	Synchronization, Brainwave amplitude changes, PTSD symptoms reported by monthly telephone contact
	Peniston and Kulkosky, 1991	29 (15 NF vs. 14 TAU)	100	36.1 vs. 37.25 (mean)	Yes	Yes	Alpha/theta	TAU	B	MMPI-indexed personality changes Medication consumption PTSD symptoms reported by monthly telephone contact

*TAC, Thayer Activation Checklist; NF, neurofeedback condition; WL, waitlist condition; CAPS, Clinician-Administered PTSD Scale; HTQ, Harvard Trauma Questionnaire; HSCL-D, Hopkins Symptom Checklist Depression Scale; HSCL-A, Hopkins Symptom Checklist Anxiety Scale; DTS, the Davidson Trauma Scale; HAMD, Hamilton Depression Rating Scale; TOVA, Test of Variables of Attention; SMR, The sensorimotor rhythm; MMPI, Minnesota Multiphasic Personality Inventory; TAU, Treatment-as-usual*.

#### Neurofeedback Using EEG Fingerprint

EEG is mobile and low cost but with limited spatial resolution, while MRI has a high spatial resolution but with low accessibility and low cost-effectiveness. To overcome these limitations of both equipments, simultaneous EEG-fMRI was introduced to estimate the amygdala fMRI-bold signal from EEG data, which is termed the amygdala electrical fingerprint (Keynan et al., 2019). Based on this fingerprint, amygdala activity was calculated using EEG only during the neurofeedback session, which was fed back to the participants.

This procedure is applied successfully to stress management in healthy soldiers and its effectiveness was demonstrated in double blinded manner. In comparison with participants assigned to either control neurofeedback group or with no neurofeedback group, participants assigned to experimental group showed significant reduction in alexithymia and faster emotional stroop which was regarded as activating a resilience process.

### Decoded Neurofeedback (DecNef) for Fear Memory

**Figure 2:**
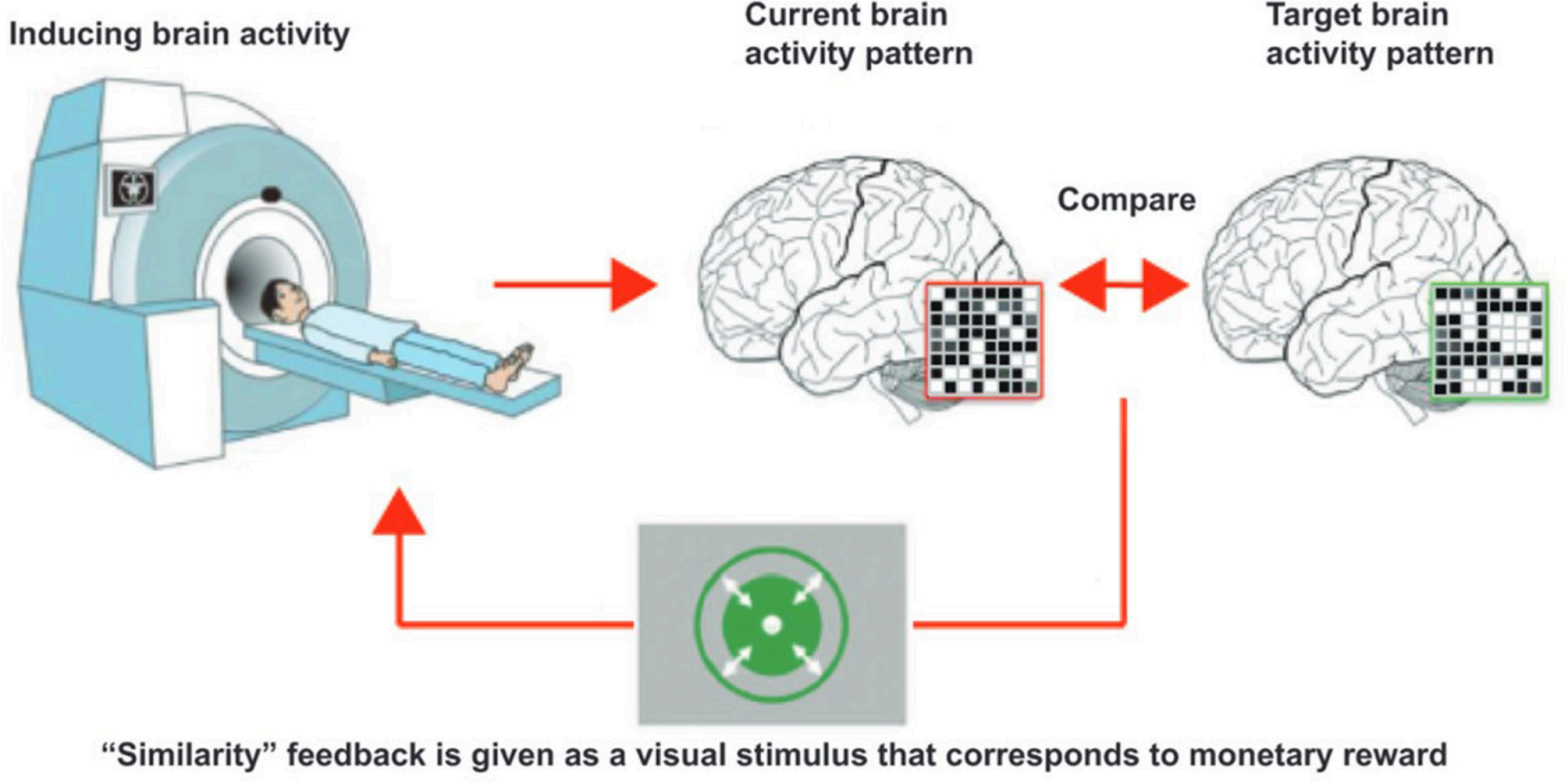
Schema of decoded neurofeedback. The participant, in the scanner, is instructed to “somehow” regulate their brain activities so that the feedback is maximized. Then, “somehow” manipulated brain activity pattern are processed as a fMRI signal and compared with target brain activity pattern. Finally, the participants are presented with a disk whose size is in proportion to the likelihood, which is also in proportion to the amount of reward the participant will gain from that trial. This cycle is then repeated. The figure is adopted from Yamada et al. (2017), with no permission required.

DecNef can be used to modify brain activity specific to different pathogeneses. Specifically, using this approach the multi-voxel activation patterns of fMRI signal within specific region of interests (ROIs) that represent designated mental experiences and states can be targeted. Figure 2 shows a conceptual schema of DecNef. Prior to DecNef training, participants first go through a fMRI decoder construction session. In this session, fMRI multi-voxel patterns for specific stimuli (e.g., red circle and green circle) are recorded. This fMRI signal is subsequently examined by a machine learning technique to decode brain activity on the basis of the presented stimuli (e.g., to decode the two fMRI signal patterns that correspond to when viewing a red and a green circle, respectively). This decoded multi-voxel pattern is used to create the target for induction in the participants brain during subsequent DecNef training in the MRI scanner (e.g., the target might be to induce brain activity related to a red, rather than a green circle). During DecNef training, real-time fMRI signal is processed immediately and the similarity between this signal and that of the target, within a predefined brain activity, is calculated online. Roughly speaking, feedback is given based on this similarity and participants aim to unconsciously and/or volitionally manipulate their own brain activity so that this similarity is increased. The feedback approximately represents the “similarity” between the target fMRI signal pattern evoked by the real stimulus (e.g., red circle or animal pictures) and a current fMRI signal pattern observed in the absence of the real stimulus. In this article, we use the term “similarity” for the sake of simplicity. Rigorously, however, the feedback is not the similarity of a current fMRI signal pattern for specific stimuli. The feedback is based on how much the decoder classifies the current fMRI signal into a target class, that is, likelihood of the target class. More concretely, the decoder was constructed to identify a stimulus (e.g., a snake picture) that is presented to a participant among different stimuli (e.g., animal pictures other than the snake) based on fMRI signal patterns. The feedback reflects the output of the decoder that represents likelihood of the target stimulus. Consequently, the feedback could be derived from hundreds or even hundreds of millions of brain activity patterns, and is an abstract index of a specified information by the decoder. This is a unique characteristic of DecNef compared with other causal methods such as optogenetics reproducing only once-occurred brain activity.

DecNef has been applied to manipulations of brain activity patterns corresponding to various mental states such as perceptual learning (Shibata et al., 2011), face preference (Shibata et al., 2016), meta-cognition (Cortese et al., 2016), color-orientation association (Amano et al., 2016), and reduction in physiological fear responses (Koizumi et al., 2016; Taschereau-Dumouchel et al., 2018).

During DecNef for reduction in physiological fear responses, participants could be trained to associate with a reward the decoded brain representation of given traumatic/distressful events. This approach might be more effective than conventional neurofeedback because it is somewhat akin to exposure therapy, which is the most effective therapy for phobia and PTSD, but does not cause the conscious awareness of the fearful event that so many people find so aversive during exposure therapy.

**Figure 3:**
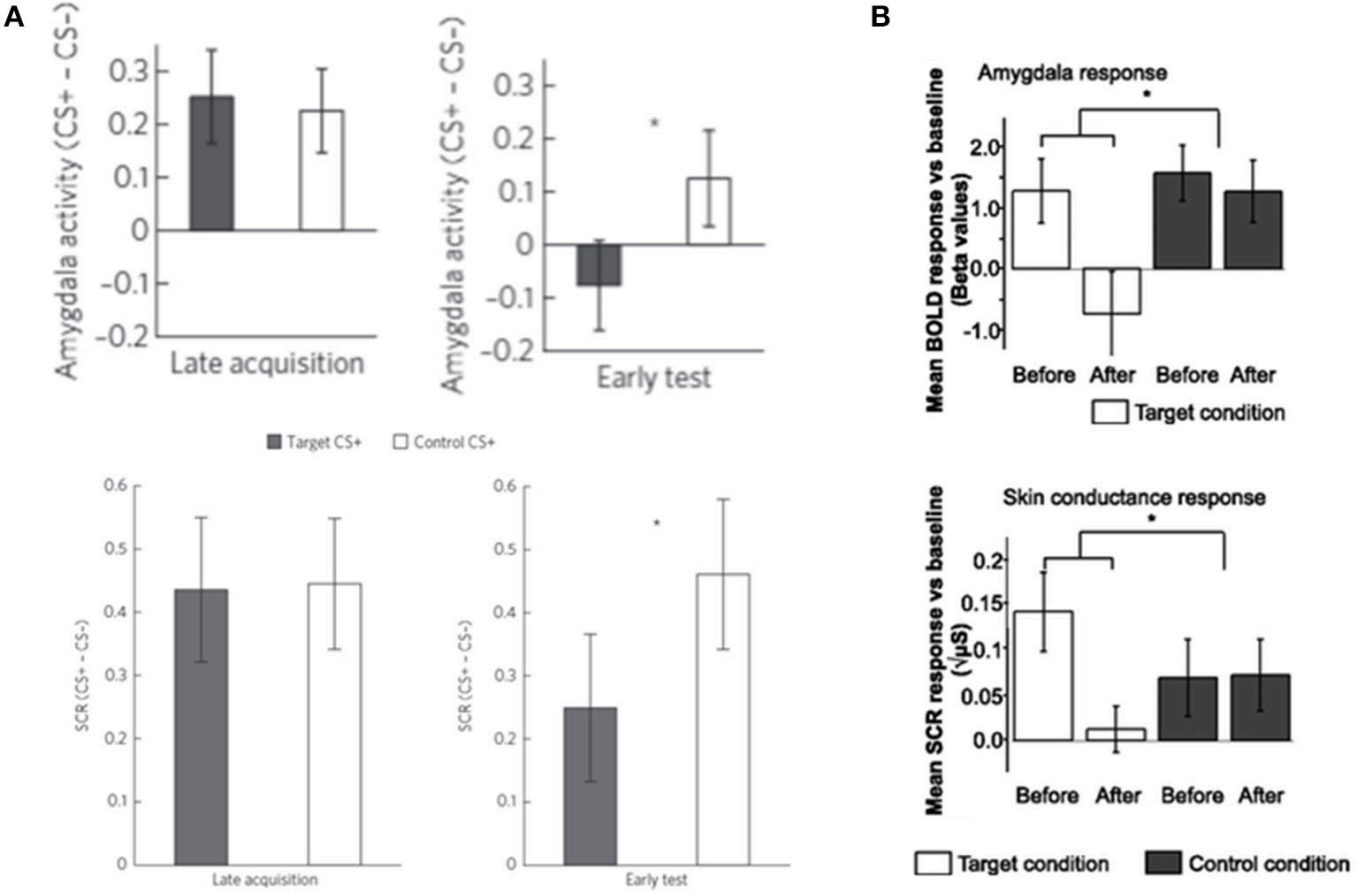
DecNef effects on fear reduction in **(A)** fear-conditioned stimuli and **(B)** feared animals. The response to target stimuli was reduce compared to control stimuli in both **(A)** fear-conditioned stimuli and **(B)** feared animals as measured by both amygdala activity and skin conductance response (SCR). Error bars represent standard errors. **(A)** Modified from Koizumi et al. (2016), with permission from the authors. **(B)** Modified from Taschereau-Dumouchel et al. (2018), with no permission required. ^*^p < 0.05.

Recent studies have shown that DecNef can reduce physiological fear responses to both fear conditioned stimuli (Koizumi et al., 2016) and feared animals (Taschereau-Dumouchel et al., 2018; Figure 3). There was particularly strong evidence for the effect of DecNef in the study with feared individuals, because this study utilized a double-blind, placebo-controlled, randomized paradigm (Taschereau-Dumouchel et al., 2018).

In the study where DecNef was used to reduce fear to fear conditioned stimuli (Koizumi et al., 2016) the multi-voxel activation pattern of activity related to the fear conditioned stimuli was paired with a reward. As a result, a significant reduction of participants' physiological fear response to these stimuli was observed. Specifically, in this study participants were told that during each trial of the DecNef training they should “somehow” self-regulate their neural activity. Unbeknown to the participants, the target was for them to induce the multi-voxel pattern of fMRI signal related to one of the two fear conditioned stimuli. On each trial, if the participant successfully induced the target pattern of fMRI signal, then they received a large reward. Thereby, via trial and error, participants learned to induce this particular pattern of neural activity, resulting in a reduced fear response to this stimulus when it was presented after DecNef training. However, this approach contains a fundamental problem for clinical application. Using this approach, prior to DecNef training, the target multi-voxel pattern of fMRI signal has to first be determined in a decoding session. This requires the explicit and repeated presentation of the target stimulus. In a laboratory setting, it is possible to decode the fMRI signal patterns for the to-be-feared conditioned stimuli a priori; i.e., ahead of fear conditioning. However, such a priori decoding is difficult in the clinical setting where patients will come in with the fear associations already strongly formed. Exposure to fear-relevant stimuli during the decoding session is likely to be highly distressful for the patients with phobia/PTSD.

This problem was overcome in a study by Taschereau-Dumouchel et al. (2018). Using a method called hyperalignment, the relevant neural representations of feared animals were inferred based on data from “surrogate” participants. Briefly, in an fMRI experiment, participants were presented with images of multiple animals and objects. In order to create the decoder of an animal feared by a designated participant, hyperalignment was used to create a “common representational space” using the neural representations of the non-fearful animals. Through this common space, it was then possible to use only the data of the “surrogate” participants to train a multi-voxel decoder of the feared animal. As such, the decoders could be trained without presenting the designated participant with aversive pictures. By subsequently using these decoders in a DecNef training, a significant reduction in the physiological fear response to the feared animals was found (Figure 3).

In summary, participants unconsciously induced brain activity for stimuli that they feared. Of importance, in contrast to conventional exposure-based therapy, these procedures evoked no distress in the participants.

#### Decoded Neurofeedback for PTSD: A Preliminary Result

**Figure 4:**
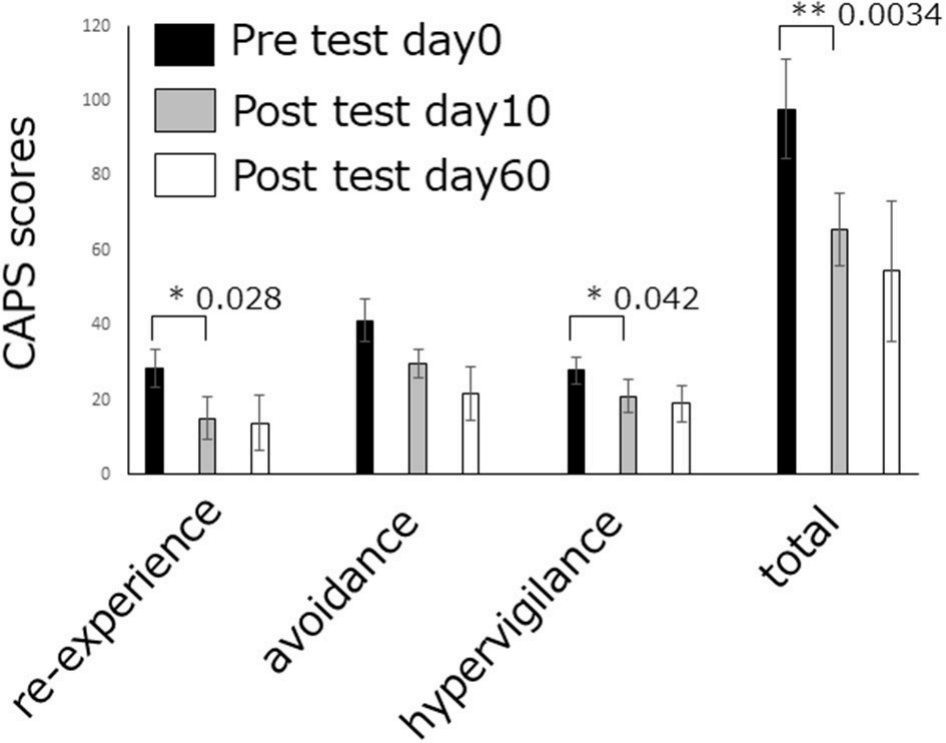
DecNef effects on PTSD amelioration. PTSD symptom cluster (i.e., re-experiencing, avoidance, and hypervigilance) and total severity scores as measured by the past week version of the CAPS-4. The re-experiencing symptoms and hypervigilance symptoms, as well as total PTSD severity at pre DecNef session reduced significantly compared at post-DecNef session. Error bars represent standard errors.

Recently, we conducted a DecNef experiment for 4 individuals with PTSD. After DecNef training, all 4 patients exhibited a clinically significant reduction (Krystal et al., 2011) (10-point decrease) in scores on the Clinician-Administered PTSD scale for DSM-4 (CAPS-4), which represents PTSD severity. Figure 4 shows the CAPS total scores before and after the intervention. After the intervention, 1 patient no longer even met the PTSD diagnosis criteria, which is defined as a total score of below 20 on CAPS (Weathers et al., 2001).

### Mechanisms of Decoded Neurofeedback (DecNef) Effect

DecNef seems to be a promising approach to treat fear-related diseases such as anxiety disorder, phobia, and PTSD. However, how DecNef reduces the fear responses is not fully understood. Two possible mechanisms have been previously postulated (Koizumi et al., 2016; Taschereau-Dumouchel et al., 2018), namely exposure-based (EB) effect and counter conditioning (CC) effect. The EB effect is consistent with the idea in conventional exposure-based therapy. That is, simple exposure to feared target under the safe condition reduces fear response to the target. This idea is also consistent with fear extinction learning. The CC effect is to change the association of the stimuli with fear by associating the stimuli with a reward (Dickinson and Dearing, 1979). That is, presentation of fearful stimuli together with reward reduces the fear response to the target. This effect is known to be larger than simple exposure effect (Newall et al., 2017).

**Figure 5:**
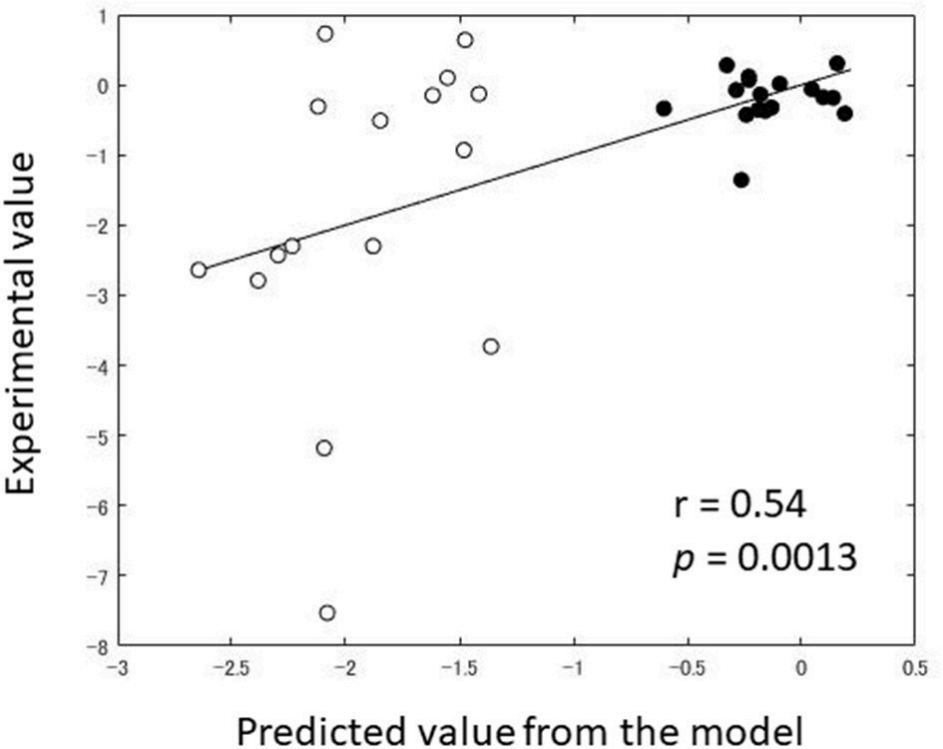
Comparison between predicted value and experimental value of Amygdala reactivity post-DecNef (target–control). Black dot indicates the individual data from Koizumi et al. (2016) while the white dot indicates the individual data from Taschereau-Dumouchel et al. (2018).

In order to dissociate the effects of EB from those of CC on fear reduction via DecNef, we mathematically modeled the DecNef effects as those derived from EB and CC separately, on the basis of Rescorla-Wagner model and synaptic plasticity rules. Briefly, we assumed that EB effect is linearly proportional to the numbers of the trials in which the target activity pattern was successfully induced (likelihood above chance). We also assumed that CC effect of each trial is proportional to the induction likelihood of the target pattern multiplied by the amount of reward, which the participant obtains the trial. This model can predict the DecNef effect (β_*EB*_ = −0.016, *p* = 0.0069, *df* = 28, β_*CC*_ = 0.014, *p* = 0.0017, *df* = 28) with a non-significant estimated intercept for the paper (1|paper = −0.692, *p* = 0.55, *df* = 28). The predicted values from the model were correlated with the experimental values (*r* = 0.54, *p* = 0.0013; Figure 5). Since negative value of *V*_*X*(*amg*)_ indicates the reduction of physiological reactivity to target stimuli, the smaller value of beta indicates that the corresponding variables are more effective. Therefore, this result suggests that EB effect, the negative coefficient, is the key component for DecNef effect on the reduction of fear response observed from Koizumi et al. (2016), and Taschereau-Dumouchel et al. (2018). The *V*_*X*(*EB*)_ and *V*_*X*(*CC*)_ have a significant effect only when data from two studies were combined. No statistically significant effect has been observed for them from a single study. With each study, the predicted values from the model were not significantly correlated with the experimental values [*r* = 0.36, *p* = 0.17 for Koizumi et al. (2016); *r* = 0.36, *p* = 0.16 for Taschereau-Dumouchel et al. (2018)], however, the effect sizes were of intermediate magnitude, in the direction expected.

## Discussion

We reviewed current status of neurofeedback trials for PTSD amelioration intended to be informative for neuroscientists in general as well as practitioners planning to use neurofeedback as a therapeutic strategy for PTSD. Despite promising results are derived from both EEG and fMRI neurofeedback (Table 1), the efficacies of these approaches have not yet been warranted.

We show preliminary data indicating that DecNef ameliorated PTSD symptoms through 3 days of feedback training. Although tentative, this result was comparable to conventional exposure therapy and conventional neurofeedback approach. Together with a short intervention period required, the results so far are encouraging to suggest that DecNef could be a promising procedure to alleviate actual PTSD symptoms. In the future, a larger sample of participants and a double-blind placebo control design are needed to demonstrate the effectiveness of this novel method for treating PTSD.

To further clarify the underlying mechanisms of DecNef, we demonstrated that the previously reported effect of DecNef in fear response reduction (Koizumi et al., 2016; Taschereau-Dumouchel et al., 2018) is estimated by the amount of successful induction of the target brain activity patterns. Whether the predominant contribution of EB effect is intrinsic to DecNef or specific to the previous two studies awaits further investigation. For example, it is worth testing the possibility that the effect of CC became noisier in the two studies because of the temporal delay of reward by several seconds. Here, we assumed that the linear term of the degree to which the targeted neural representation is successfully induced (i.e., likelihood for target pattern) corresponds to EB effects, while the quadratic term corresponds to counter conditioning effects. Although these assumptions are tentative, the results still hold that the DecNef effect in fear reduction is explained by the likelihood for successful induction of activation pattern linearly rather than by the quadratic polynomial of it. The current model should be applied to a much larger sample size for further validation in a future study.

In clinical application, DecNef has a limitation in that it can induce only specific brain activation patterns which can be decoded via multivariate pattern analysis. However, DecNef can directly access the representation for feared stimuli without eliciting conscious aversive experience if combined with procedures such as hyperalignment or CFS. This means that DecNef allows patients to be implicitly exposed to extreme traumatic stimuli with little distress, which could be advantageous to conventional exposure based therapy which can deal with only moderate traumatic stimuli.

In addition to DecNef, three promising alternative approaches have been proposed in research areas other than PTSD. First, conventional univariate fMRI-based neurofeedback can be used more effectively with deep understanding of disease. With deep understanding of Major depressive disorder, Young et al. demonstrated its efficacy utilizing a double-blind, placebo-controlled, randomized clinical paradigm (Young et al., 2017). Patients with depression show blunted amygdala hemodynamic activity to positive stimuli, and amygdala engagement appears to be critical for emotional processing and responding to both negative and positive stimuli. Based on these knowledges, they increased the amygdala's hemodynamic response to positive memories in patients with depression. Specifically, participants were instructed to retrieve positive memories while attempting to increase the hemodynamic activity in the left amygdala which was feedback to the participant as a blue bar (Young et al., 2017). This neurofeedback significantly decreased depressive symptoms and increased the percent of specific memories recalled on an autobiographical memory test. Second, EEG-fingerprint has been shown to be a feasible approach (Keynan et al., 2019). One of the fundamental problems in applications of neurofeedback for PTSD treatment arises from equipment characteristics: EEG is mobile and low cost but with limited spatial resolution, while MRI has a high spatial resolution but with low accessibility and low cost-effectiveness. To overcome these limitations, EEG-fingerprint technique enables us to estimate the amygdala fMRI-bold signal from EEG data. It can confer a participant stress resilience (Keynan et al., 2019). In the future, prospective cohort study may be needed to verify the effectiveness of this novel method for preventing PTSD development. Lastly, Functional Connectivity Neurofeedback (FCNef) (Fukuda et al., 2015; Yamashita et al., 2017) has been applied to patients of major depressive disorder and schizophrenia, and autistic participants, and its preliminary but encouraging effects have been shown (Yamada et al., 2017). Instead of brain activity patterns in specific region, FCNef manipulates the functional connectivity which is defined as synchronicity of activation between spatially apart two brain regions. FCNef allows patients to induce brain activity so as to normalize disease specific resting state functional connectivity patterns which are objectively determined using machine learning technique (Yahata et al., 2016, 2017; Yamada et al., 2017). Further development of these alternative approaches as well as of DecNef should bring more effective treatment options for wider clinical populations.

## Conclusion

In this review, we discussed recent advances in neurofeedback therapy for PTSD and presented the findings of a DecNef experiment that we conducted on patients with this disorder. While neurofeedback therapy is still in the initial stages of development, approaches such as DecNef have the potential to provide an alternative to the conventional method of PTSD treatment by preventing PTSD patients from feeling distress during the course of treatment. One limitation of this review is that since it is the dawn period of neurofeedback development, we cannot draw a conclusion from current literature what type of neurofeedback is most promising for PTSD amelioration. However, in the future, using neurofeedback approaches such as DecNef may allow for more targeted pathogenesis-based treatment of a variety of other psychiatric disorders as well.

## Author Contributions

TC, TK, AK, KI, VT-D, HL, and MK designed the research. TC, KI, and MS performed the research. TC, AK, and VT-D analyzed the data. TC, TK, AK, VT-D, SB, AH, IS, HL, HY, and MK wrote the paper.

### Conflict of Interest Statement

MK is the inventor of patents related to the DecNef method used in this study, and the original assignee of the patents is Advanced Telecommunications Research Institute International, with which the authors are affiliated. The remaining authors declare that the research was conducted in the absence of any commercial or financial relationships that could be construed as a potential conflict of interest.
